# Combined Effects of Gallic Acid Supplementation and Physical Training on Body Composition and Biochemical Parameters in Obese Patients: A Randomized, Double-Blinded, Placebo-Controlled Clinical Trial

**DOI:** 10.3390/nu18020311

**Published:** 2026-01-19

**Authors:** Bruna Kaicy Barbosa, Daniel Vinicius Alves Silva, Gislaine Candida Batista-Jorge, Berenilde Valéria de Oliveira Souza, Antônio Sérgio Barcala-Jorge, André Luiz Sena Guimarães, Alfredo Maurício Batista de Paula, João Marcus Oliveira Andrade, Sérgio Henrique Sousa Santos

**Affiliations:** 1 Laboratory of Health Science, Postgraduate Program in Health Sciences, Universidade Estadual de Montes Claros (Unimontes), Montes Claros 39401-089, MG, Brazil; 2 Postgraduate Program in Food and Health, Institute of Agricultural Sciences (ICA), Universidade Federal de Minas Gerais (UFMG), Montes Claros 39404-547, MG, Brazil

**Keywords:** obesity, gallic acid, dietary supplements, antioxidants, polyphenols

## Abstract

**Background/Objectives:** Obesity has been linked to cardiometabolic alterations and deteriorated body composition. Gallic acid, a polyphenol with antioxidant properties, may influence these parameters; however, there is limited clinical data. The aim of this study was to evaluate the effects of gallic acid supplementation combined with physical exercise in obese individuals. **Methods:** A randomized, double-blind, placebo-controlled clinical trial with 150 participants recruited and divided into eight groups according to nutritional status (eutrophic or obese), supplementation (gallic acid 200 mg/day vs. placebo), and physical exercise (trained vs. untrained) for 12 weeks. Body composition, anthropometry, and serum biomarkers were assessed before and after the intervention. Data were analyzed using repeated-measures ANOVA. **Results:** A total of 107 participants completed the final assessment. A reduction in waist-to-hip ratio was observed in the obese group trained and supplemented with gallic acid (supplement × time interaction: *p* = 0.031). There was a reduction in waist circumference (supplement × physical exercise × time interaction: *p* = 0.041) and a reduction in skinfold thickness at the pectoral (*p* = 0.044) and abdominal (*p* = 0.036) sites. Fat-free mass showed a tendency to increase in the supplemented trained obese group (*p* = 0.054). In biochemical markers, an increase in albumin was identified in the supplement × time interaction (*p* = 0.043), especially in the trained obese group. **Conclusions:** The combination of gallic acid and physical exercise promoted improvements in abdominal adiposity and body composition markers, with favorable biochemical effects.

## 1. Introduction

Obesity affects more than one billion people worldwide. Without effective control strategies, it is estimated that approximately 50% of adults will be overweight or obese by 2030 [1]. Obesity is a chronic disease that, when associated with conditions such as diabetes, dyslipidemia, and hypertension, constitutes metabolic syndrome. Metabolic syndrome is an important factor linked to increased cardiovascular morbidity and mortality [2]. Beyond its clinical implications, obesity impacts social, emotional, and economic domains, placing a significant burden on individuals and healthcare systems.

The management of obesity involves weight reduction and modulation of metabolic parameters that are essential for preventing cardiometabolic diseases. Lifestyle modifications, such as a balanced diet and regular exercise, form the basis of treatment. However, the complex aetiology of obesity, involving genetic, environmental and behavioural factors, means that these interventions alone are not always effective, contributing to the continued rise in global prevalence [3,4]. In this context, identifying compounds that can enhance metabolic responses is a critical area of research [5,6,7,8].

Interest in natural dietary supplements as an adjunct to non-pharmacological strategies has been growing. Among the substances studied, polyphenols are a particularly promising class. There is evidence that compounds such as resveratrol, curcumin and gallic acid can significantly impact the management of obesity and metabolic syndrome [9,10,11,12]. These compounds exert their effects through multiple mechanisms, including enhancing thermogenesis, inhibiting adipogenesis, improving insulin sensitivity, and modulating lipid metabolism [13].

Gallic acid, a polyphenol found in various polyphenol-rich foods and widely distributed in plants, has gained prominence as a biologically relevant metabolite characterised by well-established antioxidant and anti-inflammatory properties. In animal models, gallic acid has demonstrated the potential to modulate lipid metabolism by activating AMP-activated protein kinase (AMPK), thereby reducing body weight and attenuating hepatic steatosis [12,14]. Furthermore, hypoglycaemic effects have been reported, which are associated with the stimulation of insulin secretion by beta cells and enhanced insulin sensitivity [15], as well as increased thermogenesis, which is mediated by the activation of sirtuin 1 (SIRT1) in brown adipose tissue [16].

When combined with physical exercise, gallic acid may also improve performance metrics and muscle recovery. Animal studies have shown reduced fat accumulation, enhanced antioxidant capacity, and attenuation of mitochondrial oxidative stress, promoting muscle mass gain and greater metabolic flexibility during exercise [17,18,19].

Despite this promising preclinical evidence, clinical trials evaluating gallic acid supplementation for obesity management are lacking. Given that other polyphenols have been extensively studied in humans, investigating gallic acid offers an opportunity to explore a biologically relevant compound yet to be clinically evaluated. Therefore, this study aimed to assess, for the first time in humans, the effects of gallic acid supplementation combined with physical exercise in obese adults. The central hypothesis was that gallic acid supplementation, particularly when paired with physical exercise, would yield beneficial effects on body composition and metabolic outcomes in this population.

## 2. Materials and Methods

### 2.1. Participants

Sample size estimation was performed a priori using G*Power software (version 3.1.9.7; Universität Kiel, Kiel, Germany), based on a repeated-measures ANOVA model with within–between subject interaction [20]. The calculation assumed a medium effect size (η^2^ = 0.059; f = 0.25), in accordance with Cohen’s (1988) criteria [21] and supported by evidence from a methodologically comparable study [22].

To minimize the risk of type I error arising from multiple comparisons, a conservative significance level was adopted (α = 0.01) [23]. Assuming a statistical power of 80% (1 − β) [23], eight experimental groups, two repeated measurements, and an estimated within-subject correlation of 0.5, the analysis indicated a minimum required sample size of 96 participants. To ensure analytical robustness and account for potential attrition, a total of 150 individuals were recruited in Montes Claros, Minas Gerais, Brazil, between October 2024 and April 2025, using online advertisements and printed materials.

Eligible participants were men and women aged 18–35 years, classified as sedentary (no regular physical exercise for at least three months) and apparently healthy, as determined by the Physical Activity Readiness Questionnaire (PAR-Q). Exclusion criteria included musculoskeletal injuries, use of anti-inflammatory drugs, analgesics, or medications for obesity treatment, as well as pregnancy and lactation.

### 2.2. Experimental Design

This study was a double-blind, randomized, placebo-controlled clinical trial. The primary outcome of this exploratory trial was changes in body composition, with emphasis on central adiposity. After assessing Body Mass Index (BMI) and body composition, participants were stratified into two groups: eutrophic, defined as BMI ≤ 25 kg/m^2^, and overweight/obese, defined as BMI ≥ 25 kg/m^2^ associated with a body fat percentage greater than 25%. BMI was calculated by dividing body weight (kg) by height squared (m^2^). Randomization was performed by a second evaluator, unrelated to the collection and analysis stages, randomly allocating each participant to one of eight subgroups formed by the combination of factors: nutritional status (eutrophic or obese), intervention (placebo or gallic acid), and level of physical activity (trained or untrained). Blinding was maintained for participants, researchers responsible for measurements, and laboratory staff.

Participants preselected by telephone were invited to Clemente de Faria University Hospital to confirm eligibility criteria. At this initial meeting, a detailed explanation of the experimental protocol was provided, and participants signed the Informed Consent Form (ICF). The PAR-Q was then administered. Individuals allocated to the trained groups performed strength training four times a week. The intervention lasted 12 weeks, with blood collection and body composition assessment before and after the experimental period.

### 2.3. Supplement

Gallic acid was provided to participants in the form of capsules containing 200 mg of the pure compound. The capsules were purchased from the Exodo Científica laboratory and handled in accordance with the Good Practices for the Handling of Magistral and Official Preparations for Human Use standards (Brazil, 2007) [24]. The placebo capsules were identical in size and color and contained only starch. The supplement was delivered monthly in bottles containing 30 capsules, for a total of three months’ worth. Participants were instructed to take one capsule per day.

### 2.4. Nutritional Counseling

All participants received standardized nutritional guidance, provided in tabular format. The meal plan was based on Total Energy Expenditure (TEE) and categorized into three calorie ranges (Diet A, B, and C). To ensure homogeneity between groups, macronutrient distribution was standardized across the three diets. Additionally, participants were instructed to avoid foods rich in gallic acid and to abstain from alcohol consumption throughout the study.

### 2.5. Physical Training

Exercise sessions were supervised by qualified professionals. Training progression was predefined in the study protocol and implemented through increases in perceived training load every four weeks. Groups assigned to physical exercise underwent 50 min of strength exercises and 20 min of aerobic activity four times a week for 12 weeks. These exercises consisted of three sets of ten repetitions of leg curls, leg extensions, and leg abductions; front pulls; chest flyes; triceps pulleys; and barbell curls, with a one-minute interval between each set [25].

### 2.6. Body Composition

Body composition was assessed using tetrapolar bioimpedance (BIA1010, Sanny®, São Paulo, SP, Brazil) and by measuring skinfolds with a Cescorf (Porto Alegre, RS, Brazil) clinical adipometer. These assessments were conducted after participants fasted for at least three hours and were wearing light clothing, had an empty bladder, and were free of metallic objects. For the bioimpedance assessment, participants remained supine with their arms extended at their sides and their legs apart, and the equipment was calibrated beforehand. Skin fold measurements were performed with the individual in an upright position and their upper limbs relaxed. Weight and height were obtained using a calibrated Balmak electronic scale (BK-50FA, Balmak®, Santa Bárbara d’Oeste, SP, Brazil) by the Institute of Weights and Measures. Waist and hip circumferences were measured using a non-elastic tape measure with 0.5 cm graduations, positioned at the smallest circumference between the last rib and the iliac crest (waist) and at the largest gluteal prominence (hip). The waist-to-hip ratio (WHR) was calculated by dividing the waist measurement by the hip measurement.

### 2.7. Laboratory Tests

After fasting for 12 h, blood was collected by peripheral venous puncture in the morning at Clemente de Faria University Hospital. Tests were performed in an accredited laboratory using ELISA enzyme kits (DSA BioELISA) for fasting glucose, glycated hemoglobin (HbA1c), aminotransferase (ALT), aspartate transaminase (AST), insulin, complete blood count, total cholesterol, high-density lipoprotein (HDL-c), low-density lipoprotein (LDL-c), triglycerides (TG), creatinine, urea, and albumin. All analyses were performed at two time points: pre-intervention and post-intervention.

### 2.8. Adherence and Adverse Events

Throughout the intervention, participants were contacted weekly by telephone to monitor adherence and identify possible adverse events, which were systematically recorded. Participants who did not adhere to the protocol were discontinued from the study. At the end of 12 weeks, volunteers completed a standardized questionnaire to assess adverse reactions.

### 2.9. Ethical Aspects

The study was approved by the Research Ethics Committee of the State University of Montes Claros (Unimontes) (opinion no. 4,735,915) on 26 May 2021, and registered in the Brazilian Registry of Clinical Trials (ReBEC) under number RBR-7st4f9b. It was conducted in accordance with the ethical principles of the Declaration of Helsinki. All participants provided free and informed consent.

### 2.10. Statistical Analysis

The data were analyzed using Statistical Package for Social Science (SPSS) version 25 (IBM Corporation, Armonk, NY, USA), and the graphs were prepared using GraphPad Prism version 8.0. The normality of the distributions was assessed using the Shapiro–Wilk test. The baseline characteristics of the participants were presented descriptively: continuous variables with normal distribution were expressed as mean ± standard deviation, while non-normally distributed variables were described as median and interquartile range (IQR). Categorical variables were presented as absolute and relative frequencies (%). To assess the effects of the intervention, repeated-measures ANOVA with a mixed design was used, considering (i) the effect of time (intragroup); (ii) the effect of the group (intergroups); and (iii) the time × group interaction, which indicates whether the change in variables throughout the intervention differed between groups. In addition, intragroup comparisons (pre vs. post) were performed using the paired *t*-test when the distribution was normal. The level of significance adopted was *p* < 0.05, and the effect size was estimated by partial eta squared (η^2^p), as provided by SPSS. Comparisons between groups were performed using ANCOVA with baseline as a covariate. Paired comparisons of estimated marginal means were adjusted using the Bonferroni method.

## 3. Results

### 3.1. Participant Flow and Baseline Characteristics

**Figure 1:**
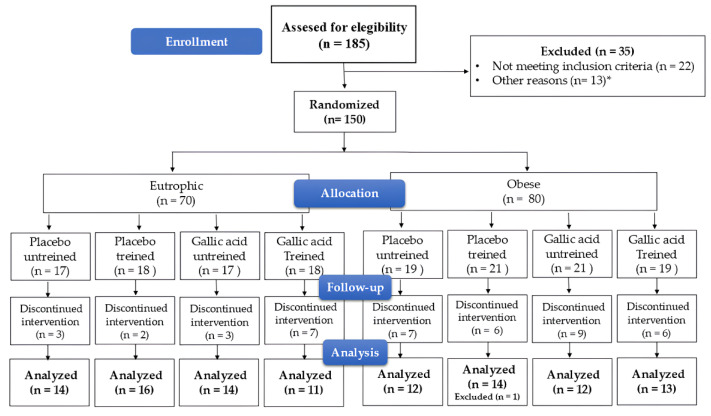
Diagram consort. * Other reasons: 10 withdrew from the study; 2 relocated to another city; 1 required surgery.

Between October 2024 and April 2025, a total of 106 participants were included in the final analysis. One participant was excluded due to the development of extreme values during the study period. The exclusion was based on predefined outlier criteria and was not related to treatment response or study outcomes. Sensitivity analyses including this participant were conducted (see Supplementary Materials). The distribution among the eight experimental groups corresponded to the planned randomization scheme. The study completion rate was high: 71.3% of participants completed the 12-week intervention, with similar proportions between the placebo and gallic acid groups, as well as between the trained and untrained groups. A total of 43 participants discontinued the intervention. Additional data on participant flow, inclusion, exclusion, and representativeness are presented in Figure 1.

Demographic, anthropometric, biochemical, and body composition characteristics were broadly balanced between groups at baseline (Table 1).

The median age of the 106 participants was 25.0 years (IQR: 22.0–29.3) and ranged from 22 to 31.5 years across the different groups, reflecting the target population of young adults. Most participants were female (67% in total, varying by group). Body weight and BMI showed the expected pattern: the eutrophic groups had a BMI between approximately 20.2–21.7 kg/m^2^ and the average weight of the participants was 59.7 kg, while the overweight/obese groups had BMIs typical of obesity (30.0–32.7 kg/m^2^) and an average weight of 90.4 kg (Table 1).

Waist and hip circumferences, as well as waist-to-hip ratio (WHR), reflected clear differences between eutrophic and obese individuals, according to the study design. Body composition measurements obtained by bioimpedance and skinfold thickness also followed this pattern, with higher fat mass values and lower relative muscle mass proportions in the obese groups. Biochemical parameters showed baseline values within the expected ranges for apparently healthy young adults. The clinically relevant changes observed were mainly restricted to the lipid profile, with high LDL-c and triglyceride values and reduced HDL-c levels, predominantly in the obese groups.

### 3.2. Body Composition

Changes in body composition, assessed using anthropometric measurements, skinfold thickness, and bioimpedance, are presented in Table 2 and Table S2. An interaction between supplementation and time was observed in the waist-to-hip ratio (*p* = 0.031; ES = 0.048), characterized by a reduction in the trained obese group supplemented with gallic acid (0.79 [CI: 0.78–0.81] to 0.78 [CI: 0.766–0.801]), while the trained obese group receiving placebo showed no changes (0.79 [CI: 0.77–0.80] to 0.79 [CI: 0.772–0.810]). In addition, the four-way interaction between supplement, physical exercise, BMI, and time for waist circumference showed a trend toward significance (*p* = 0.076, ES = 0.036), attributed to the reduction in the trained obese group and the absence of changes in the other groups. Anthropometric measurements did not reveal significant interactions in relation to weight, BMI, and hip circumference.

Regarding lean mass, the data indicated a trend toward interaction between supplementation, physical exercise, BMI, and time (*p* = 0.054; ES = 0.037), with a slight increase observed in trained obese participants who received gallic acid (51.83 [CI: 46.75–55.43] to 52.92 [CI: 48.41–57.42]), while a reduction was observed in the placebo group (52.02 [CI: 47.63–56.41] to 51.09 [CI: 46.75–55.42]). In addition, skinfold measurements reinforced these findings, showing a significant interaction for the pectoral skinfold (*p* = 0.044, ES = 0.044) and an interaction between supplementation and time for the abdominal skinfold (*p* = 0.036, ES = 0.044). In both cases, a more pronounced reduction in skinfolds was observed in obese participants supplemented with gallic acid when compared to the placebo group (Table 2).

**Figure 2:**
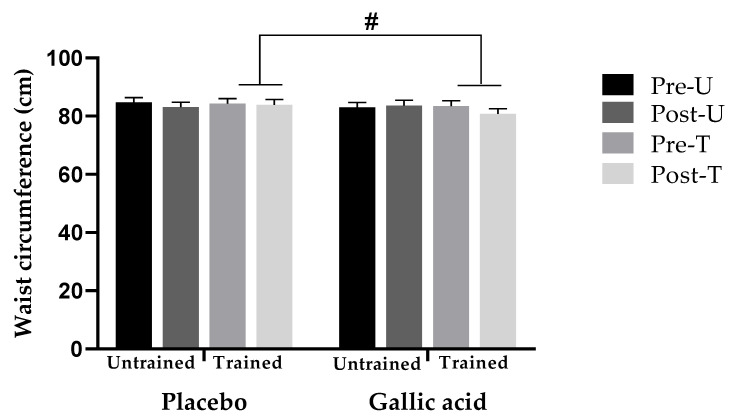
Changes in waist circumference. Values are expressed as the mean. # Significant interaction between physical activity × supplementation × time at *p* < 0.05.

Additionally, an interaction between physical exercise, supplement, and time was observed in waist circumference (*p* = 0.041, ES = 0.047), with a reduction observed in the trained group supplemented with gallic acid (83.47 [CI: 79.62–87.31] to 81.77 [CI: 77.91–85.64]), and no change observed in the trained placebo group acid (83.55 [CI: 79.99–86.79] to 83.23 [CI: 79.66–86.79]) (Figure 2).

### 3.3. Hematological and Biochemical Parameters

Hematological analysis did not reveal significant differences throughout the study, remaining within clinical reference ranges, and comparisons between groups are presented in Supplementary Table S3. In biochemical analyses, an interaction between supplementation and time was observed in insulin levels (*p* = 0.043, ES = 0.043). This interaction occurred due to a decrease in insulin in the placebo group that performed physical activity (9.78 [CI: 8.07–11.47] to 7.42 [CI: 5.76–9.09]), in contrast to the maintenance of levels in the gallic acid group. A significant interaction between supplementation and time was also identified in relation to albumin (*p* = 0.043, ES = 0.043), showing an increase in levels in the group of trained obese individuals supplemented with gallic acid (Table 3). For the other biochemical variables, no significant interactions involving supplementation and time were identified, nor was there any effect on the interaction between supplementation, physical exercise, BMI, and time.

**Figure 3:**
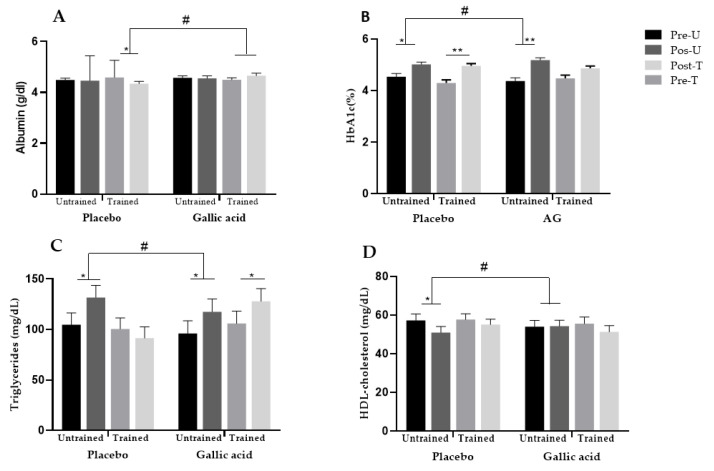
Changes in biochemical parameters (**A**) Albumin; (**B**) glycated hemoglobin (HbA1c); (**C**) triglycerides; (**D**) HDL-cholesterol. * Significantly different within the group at *p* < 0.05; ** significantly different within the group at *p* < 0.01; **#** Significant interaction between physical activity × supplementation × time at *p* < 0.05.

In the analysis of the interaction between supplementation, physical exercise, and time, significant effects were identified when comparing the placebo and gallic acid groups in the biochemical parameters. A significant interaction was observed for albumin (*p* = 0.047; ES = 0.041). Specifically, the trained group supplemented with gallic acid showed a slight increase in albumin levels throughout the experimental period acid (4.50 [CI: 4.35–4.64] to 4.65 [CI: 4.45–4.85]), while the trained group that received a placebo showed a significant reduction from pre- to post-intervention (*p* = 0.036) (4.59 [CI: 4.46–4.72] to 4.34 [CI: 4.16–4.53]) (Figure 3A).

Regarding glycemic control, glycated hemoglobin showed greater variation in the trained group supplemented with gallic acid compared to the placebo group (*p* = 0.021; ES = 0.054). In addition, a significant increase was observed between the pre- and post-intervention periods in all groups (*p* < 0.05) (Figure 3B). Furthermore, an interaction between physical exercise, supplementation, and BMI indicated that the effect of gallic acid on glucose reduction was more pronounced in obese individuals who performed physical exercise, with no relevant changes in the other groups (*p* = 0.031; ES = 0.049).

In relation to the lipid profile, triglyceride levels increased significantly in all groups except the trained group that received a placebo. Among untrained individuals, the placebo group showed a more pronounced increase when compared to the group supplemented with gallic acid (*p* = 0.038; ES = 0.045) (Figure 3C). Similarly, among untrained individuals, a reduction in HDL-c levels was observed in the placebo group, while no significant changes were detected in the group supplemented with gallic acid (*p* = 0.050; ES = 0.040) (Figure 3D).

### 3.4. Safety Profiles

No serious adverse reactions were observed during the study, and no participants withdrew from the intervention due to unwanted effects. The adverse reactions that did occur were mild and distributed similarly between the placebo (37.5%) and gallic acid (46.9%) groups. The most frequent reactions in the placebo group were excessive sleepiness (8.9%), insomnia (5.4%) and diarrhea (5.4%). In the gallic acid group, comparable proportions of excessive sleepiness (10.2%), insomnia (6.2%) and diarrhea (4.2%) were observed. Other reactions that were reported more frequently were pimples (6.1%) and skin redness (8.2%), though there was no statistical difference compared to the placebo group. The other reactions, headache, polyuria, palpitations, dry lips, and abdominal pain, occurred less frequently in both groups.

## 4. Discussion

This study is the first to investigate the effects of combining pure gallic acid supplementation with physical activity in young obese adults. The main findings suggest that, when combined with physical training, gallic acid supplementation improves the waist-to-hip ratio by reducing abdominal circumference and increasing lean mass, while decreasing abdominal and pectoral skinfolds. Biochemically, an interaction between supplementation and time was observed, as evidenced by increased albumin levels in the trained obese group compared to the placebo group.

The waist-to-hip ratio (WHR) is an important parameter used to assess the risk of cardiometabolic diseases, as it is directly related to harmful abdominal fat, which is associated with conditions such as type 2 diabetes mellitus and high blood pressure [26]. Evidence shows that the WHR is strongly correlated with the fat index, making it a useful risk assessment tool, particularly since BMI has limitations in determining obesity [27,28,29]. In the present study, a reduction in WHR was observed in obese individuals who took gallic acid; this effect was not seen in the group who exercised and took a placebo. This suggests that the improvement may be due to gallic acid supplementation. This finding is particularly relevant as it indicates a potential reduction in these individuals’ cardiometabolic risk.

The reduction in WHR is consistent with the other findings of this study. While no significant changes were observed in the other body composition parameters, a decrease in waist circumference and abdominal skinfold thickness was noted, suggesting a favourable change in fat distribution. This is consistent with previous research, which also observed a reduction in waist circumference in overweight women who consumed a cocoa-based drink containing 69.24 mg of gallic acid per serving [30].

Another notable outcome of this study was the observed increase in lean mass among individuals in the trained obese group. Current weight loss treatments include glucagon-like peptide-1 (GLP-1) and glucose-dependent insulinotropic polypeptide (GIP) receptor agonists. While these drugs are effective, they have the disadvantage of causing lean mass loss. Therefore, the search for strategies that can help maintain lean mass in patients undergoing obesity treatment remains a challenge [31]. A previous in vitro study with gallic acid, using primary mouse myoblasts, demonstrated its ability to promote myogenic differentiation. When administered to mice, gallic acid promoted a significant increase in muscle mass without altering body weight or other metabolic parameters, a finding consistent with that observed in the present study [19].

The effects of gallic acid on muscle function are due to its anti-inflammatory and antioxidant properties. These properties give gallic acid the ability to modulate oxidative stress and preserve mitochondrial function after exercise. In this way, gallic acid can create a physiological environment that is more conducive to muscle tissue repair and protein synthesis. This reduces muscle damage and inflammation, thereby increasing fat-free mass [17].

Animal studies show that gallic acid has promising effects on metabolic parameters, including improvements in hepatic steatosis, blood glucose and lipid profiles [12,15,32]. However, analyses that considered stratification of groups by BMI showed no significant changes in these metabolic variables. Nevertheless, analyses performed by stratifying participants only by supplementation aimed to assess the impact of the interaction between supplementation, physical exercise and time, regardless of body composition, showed effects on biochemical measures.

This interaction may reinforce the effect of the supplement on serum albumin, since an increase was also observed in the trained group. An increase in albumin within clinical limits in healthy young people, as observed in this study, has been associated with an increase in lean body mass in various contexts, and may indicate improved muscle recovery, particularly following physical exercise [33]. This finding contrasts with the maintenance of values in the placebo group, suggesting possible interference from the supplement, although other factors cannot be ruled out. Furthermore, higher albumin levels have been linked to more favourable cardiovascular outcomes, given that reduced concentrations of this protein are associated with coronary artery disease or heart failure [34]. Similarly, a previous clinical study involving epigallocatechin gallate supplementation, a polyphenol whose structural components include gallic acid, also observed an increase in albumin only in the treated group. This suggests a consistent response pattern, although the pathophysiological relevance of this effect in humans should still be interpreted with caution [35].

Our findings diverge somewhat from those derived from animal models in the literature, which often describe the beneficial effects of gallic acid on glucose and lipid metabolism parameters. In the present study, however, no reduction in these markers was observed. On the contrary, an increase in glycated haemoglobin and triglycerides was observed in all groups, regardless of supplementation. However, significant interactions between supplementation, physical exercise and time were only observed in untrained individuals. Additionally, no significant differences were detected between the pre- and post-intervention periods among trained participants who received gallic acid supplementation, unlike in the other groups. This pattern may indicate that the combination of physical exercise and supplementation was associated with a reduction in these metabolic markers. Regarding HDL-c, a significant reduction was observed in the trained group that received a placebo, while the trained group that received gallic acid supplementation showed no changes throughout the intervention period. These results suggest a possible modulatory role of gallic acid on this lipid variable, though they do not establish a direct causal relationship.

This study has some limitations that should be considered when interpreting the results. Participants did not receive a standardised diet, which meant that caloric and nutritional intake could not be strictly controlled; only general dietary guidelines were provided. Additionally, while the a priori sample size calculation was met, the relatively high dropout rate, combined with the absence of an intention-to-treat analysis, resulted in a small final participant number, which may have limited the statistical power to detect more significant effects. Another limitation is the relatively short intervention period, which may not have been sufficient to fully capture metabolic adaptations.

Despite these limitations, this is a feasibility study in which statistically robust effects are not necessarily expected. Nevertheless, observing significant results and considerable effect sizes demonstrates the potential impact of the intervention and reinforces the need to design subsequent clinical trials with greater statistical power and a longer duration to validate and deepen the interpretation of these results. Furthermore, dose–response assessments and the inclusion of mechanistic outcomes could clarify the clinical relevance and sustainability of gallic acid supplementation, particularly in obese populations.

## 5. Conclusions

The findings of this randomized clinical trial indicate that gallic acid supplementation, particularly when combined with regular physical exercise, promotes selective benefits in young adults with obesity. Reductions in waist-to-hip ratio and waist circumference were observed, as well as more pronounced decreases in pectoral and abdominal skinfolds, especially among trained obese individuals who received the supplement. There were also indications of an increase in fat-free mass and albumin levels in this same subgroup, suggesting possible improvement in muscle recovery and a more favorable metabolic profile. Although these results are promising, the relatively short intervention period and sample size limit the generalization of the findings. Thus, longer-term studies with larger samples and more rigorous nutritional control are needed to confirm and deepen the understanding of the effects of gallic acid in different populations.

## Figures and Tables

**Table 1:** Demographic and Clinical Characteristics of the Participants at Baseline.

Characteristic	Placebo (*n* = 56)				Gallic Acid (*n* = 50)			
	Eutrophic (U) (*n* = 14)	Eutrophic (T) (*n* = 16)	Obese(U) (*n* = 12)	Obese (T) (*n* = 14)	Eutrophic (U) (*n* = 14)	Eutrophic (T) (*n* = 11)	Obese (U) (*n* = 12)	Obese (T) (*n* = 13)
Demographics								
Age, years ^a^	22 (19–25)	22 (21–25.50) *	31.5 (28.5–34.5)	27 (23–32)	24.5 (23–28)	22 (20.5–25.5) *	27 (24–29)	28 (24–31)
Female sex—no. (%)	7 (50.0)	11 (68.8)	9 (75)	11 (78.6)	10 (71.4)	5 (45.5)	9 (75.0)	9 (69.2)
Anthropometry								
Body weight (kg) ^b^	61.0 ± 9.5	57.3 ± 9.5	86.9 ± 8.9	92.4 ± 18.9	60.2 ± 8.5	60.9 ± 11.9	90.5 ± 16.8	91.3 ± 16.5
Body mass index (kg/m^2^) ^a^	21.6 (18.6–22.6)	20.2 (19.4–24.2)	32.7 (29.4–34.8)	32.3 (30.8–37.7)	21.7 (19.4–24.2)	21.7 (19.5–22.9)	32.4 (28.2–37.6)	30.0 (29.0–40.7) *
Waist circumference (cm) ^b^	71.5 ± 6.8	69.6 ± 6.9	97.9 ± 9.4	96.0 ± 11.5	70.3 ± 6.7	71.6 ± 8.9	95.0 ± 8.6	96.6 ± 10.3
Hip circumference (cm) ^a^	96.5 (90.0–98.0)	92.5 (86.5–96.0)	109.0 (104.0–121.5)	110.0 (113.5–126.0)	97.0 (89.0–98.0)	96.5 (90.0–98.7)	117.0 (105.0–124.5)	109.0 (107.0–131.5) *
Waist-to-hip ratio ^a^	0.76 (0.72–0.79)	0.74 (0.72–0.79)	0.81 (0.79–0.85) *	0.83 (0.81–0.85)	0.73 (0.70–0.77)	0.74 (0.70–0.79)	0.83 (0.81–0.85)	0.83 (0.77–0.87)
Body Composition								
Fat-free mass (kg) ^a^	44.6 (36.3–49.9)	41.3 (37.3–46.7) *	51.5 (49.7–56.2)	48.4 (45.4–58.2)	41.1 (37.8–43.7) *	40.4 (36.9–51.4)	49.2 (46.4–54.6) *	51.6 (43.3–58.4)
Skeletal muscle mass (%) ^a^	39.9 (31.4–43.1)	36.7 (32.5–43.5)	28.3 (24.1–31.9)	26.6 (24.7–28.7) *	34.5 (32.0–39.5)	37.2 (31.8–39.1)	27.8 (26.0–32.2)	25.8 (25.1–31.6)
Body fat (kg) ^b^	16.7 ± 5.6	14.7 ± 6.3	35.0 ± 9.5	40.4 ± 12.6	17.8 ± 6.7	16.4 ± 5.4	37.8 ± 9.7	39.5 ± 13.4
Biochemistry/Lipid level								
Glucose (mg/dL) ^a^	82.5 (72.0–84.0)	76.0 (72.5–79.5)	76.0 (73.5–82.0)	80.0 (76.0–86.0)	79.5 (73.0–84.0)	79.0 (72.0–88.0) *	82.0 (77.5–90.0)	76.0 (73.5–81.0)
Glycated hemoglobin (%) ^a^	4.80 (4.60–5.00)	4.55 (3.75–4.95)	4.45 (4.00–4.70)	4.40 (4.10–4.60)	4.85 (4.10–5.10) *	5.00 (4.90–5.20) *	3.95 (3.80–4.65)	3.90 (3.75–4.30)
Insulin (µIU/mL) ^a^	6.60 (4.60–7.50)	4.50 (3.05–7.55)	10.00 (7.80–15.00)	10.90 (9.80–14.30) *	4.65 (2.30–7.20)	4.60 (3.85–6.10)	14.10 (11.55–18.75)	11.10 (8.00–13.70)
Urea (mg/dL) ^a^	21.0 (26.5–30.0)	22.0 (19.0–27.5) *	26.5 (20.0–34.5)	24.5 (20.0–31.0)	24.0 (22.0–310)	27.0 (23.0–31.0)	23.5 (19.0–31.5)	24.0 (20.5–27.5)
Creatinine (mg/dL) ^b^	0.92 ± 0.25	0.83 ± 0.23	0.90 ± 0.21	1.01 ± 0.17	0.90 ± 0.21	0.94 ± 0.21	0.92 ± 0.12	0.97 ± 0.13
Albumin (g/dL) ^a^	4.65 (4.40–4.90)	4.60 (4.60–4.80)	4.35 (4.10–4.60) *	4.54 (4.30–4.70)	4.70 (4.60–4.90)	4.80 (4.75–4.90) *	4.50 (4.20–4.60)	4.50 (4.10–4.60) *
AST (U/L) ^a^	20.0 (16.0–21.0) *	17.0 (16.0–20.5)	17.0 (16.0–25.0) *	18.0 (14.0–26.0)	19.0 (16.0–20.0)	24.0 (19.5–29.0) *	22.0 (19.0–28.0)	19.0 (18.0–22.0)
ALT (U/L) ^a^	15.0 (13.0–17.0) *	13.5 (11.0–16.5) *	20.0 (17.0–26.0) *	18.5 (12.0–33.0) *	15.0 (13.0–23.0) *	21.0 (14.5–32.0)	35.0 (25.0–42.5)	21.0 (19.0–28.0) *
Uric acid (mg/dL) ^a^	4.10 (3.10–5.10)	3.35 (3.10–3.75) *	4.45 (2.85–5.30)	4.40 (3.90–4.50)	3.70 (2.70–4.60)	3.10 (2.90–4.80)	4.70 (3.50–5.90)	5.00 (4.30–6.00)
Total cholesterol (mg/dL) ^a^	161.0 (138.0–199.0)	146.5 (130.0–159.5) *	200.5 (173.0–250.0)	195.0 (169.0–212.0)	159.0 (146.0–193.0)	178.0 (163.0–225.0)	178.5 (134.5–221.0)	181.0 (136.0–198.0)
HDL cholesterol (mg/dL) ^a^	63.0 (50.0–82.0) *	61.5 (50.5–75.5)	42.0 (37.5–53.0) *	48.5 (42.0–58.0) *	58.0 (48.0–62.0)	65.0 (55.5–70.5)	48.0 (41.5–52.5) *	42.0 (34.0–49.0)
LDL cholesterol (mg/dL) ^a^	85.0 (64.0–102.4)	74.5 (57.0–83.4) *	121.5 (107.6–173.0)	108.5 (98.0–129.0)	85.0 (75.0–110.0)	101.0 (80.0–137.0)	108.0 (77.5–140.5)	99.0 (58.0–127.0)
Triglycerides (mg/dL) ^a^	74.5 (53.0–91.0)	57.0 (45.5–85.5) *	141.5 (89.5–162.0)	99.0 (73.0–121.0) *	64.0 (57.0–74.0)	80.0 (58.0–119.5)	98.0 (80.5–151.5) *	128.0 (79.0–153.0)

* Non-normal distribution according to the Shapiro–Wilk test (*p* < 0.05); ^a^ Median (IQR); ^b^ Mean ± SD; U: Untrained; T: Trained.

**Table 2:** Effects on body composition before and after 12 weeks of intervention.

	Placebo (*n* = 56)								Gallic Acid (*n* = 50)											
	Eutrophic (U) (*n* = 14)		Eutrophic (T) (*n* = 16)		Obese(U) (*n* = 12)		Obese (T) (*n* = 14)		Eutrophic (U) (*n* = 14)		Eutrophic (T) (*n* = 11)		Obese (U) (*n* = 12)		Obese (T) (*n* = 13)		Supplement × PE × BMI × Time Interaction		Supplement × Time Interaction	
Parameter	Pre	Post	Pre	Post	Pre	Post	Pre	Post	Pre	Post	Pre	Post	Pre	Post	Pre	Post	*p*	ES	*p*	ES
Anthropometry																				
BW (kg)	60.97 (9.51)	61.83 (9.21)	57.29 (9.55)	58.03 (9.72)	86.92 (8.86)	86.12 (7.91)	92.43 (18.86)	90.40 (15.54)	60.18 (8.49)	61.16 (7.61)	60.90 (11.93)	61.67 (13.71)	90.53 (16.51)	90.40 (17.27)	91.35 (16.48)	90.53 (15.54)	0.77	0.00	0.35	0.09
BMI (kg/m^2^)	21.31 (2.35)	21.54 (2.50)	20.54 (2.64)	20.75 (2.66)	32.20 (3.52)	32.82 (3.40)	34.51 (6.59)	33.99 (7.28)	21.74 (2.50)	21.86 (2.69)	21.25 (2.72)	21.52 (3.10)	32.90 (3.52)	32.69 (5.22)	33.42 (6.61)	32.96 (6.14)	0.72	0.01	0.83	0.00
WC (cm)	71.46 (6.58)	71.89 (6.87)	69.59 (6.91)	68.66 (6.57)	97.92 (9.43)	94.25 (6.90)	97.50 (13.44)	97.80 (13.67)	70.85 (6.59)	71.42 (6.18)	71.63 (8.90)	70.50 (10.89)	94.90 (9.10)	93.50 (10.60)	97.50 (13.44)	93.05 (9.66)	0.08	0.04	0.89	0.00
HC (cm)	94.78 (9.77)	95.75 (5.69)	92.16 (6.27)	93.00 (6.84)	112.45 (9.77)	112.17 (9.89)	117.93 (12.85)	117.43 (13.01)	95.27 (5.05)	95.15 (4.92)	95.00 (5.57)	94.27 (6.71)	114.95 (11.12)	115.00 (8.67)	117.17 (13.14)	116.33 (12.45)	0.94	0.00	0.28	0.01
Waist-to-hip ratio	0.75 (0.04)	0.75 (0.05)	0.75 (0.49)	0.74 (0.05)	0.83 (0.09)	0.84 (0.07)	0.81 (0.06)	0.80 (0.08)	0.74 (0.05)	0.75 (0.04)	0.75 (0.06)	0.74 (0.07)	0.83 (0.05)	0.83 (0.08)	0.87 (0.08)	0.84 (0.06)	0.90	0.00	0.03 *	0.05
Body composition																				
Fat-free mass (%)	60.18 (8.03)	72.57 (6.88)	74.78 (8.89)	57.26 (8.18)	60.18 (8.03)	61.54 (8.36)	56.82 (7.05)	57.28 (8.18)	70.97 (9.10)	70.29 (7.86)	73.39 (6.69)	72.88 (6.27)	58.49 (6.07)	58.54 (5.99)	57.46 (8.74)	59.13 (8.64)	0.18	0.01	0.53	0.01
Fat-free mass (kg)	44.30 (7.77)	43.99 (7.24)	42.61 (7.58)	43.44 (7.11)	52.44 (5.19)	52.62 (5.47)	52.02 (10.25)	51.09 (10.04)	42.39 (6.17)	42.72 (5.30)	44.46 (8.92)	43.63 (7.71)	52.73 (10.46)	52.67 (10.52)	51.82 (8.73)	52.80 (9.28)	0.05 *	0.03	0.27	0.01
Skeletal muscle mass (%)	37.53 (6.42)	36.24 (7.23)	38.29 (7.01)	36.16 (8.21)	28.52 (4.57)	32.17 (8.65)	27.45 (5.12)	27.38 (5.58)	35.17 (6.82)	34.84 (6.72)	35.17 (6.82)	36.19 (5.49)	27.45 (5.12)	28.17 (3.53)	28.15 (5.77)	28.67 (5.26)	075	0.01	0.91	0.00
Body water (%)	52.00 (5.19)	51.15 (4.84)	52.40 (5.84)	52.59 (5.26)	44.14 (5.73)	44.55 (6.70)	42.21 (5.40)	42.13 (5.91)	50.37 (4.53)	49.69 (5.18)	51.61 (4.96)	50.90 (4.80)	43.53 (4.41)	43.00 (3.66)	42.77 (5.73)	43.37 (5.13)	0.16	0.02	0.61	0.00
Body water (kg)	31.63 (5.63)	31.58 (5.45)	29.94 (5.77)	30.45 (5.47)	38.10 (3.94)	38.13 (4.83)	38.85 (8.51)	37.86 (8.87)	30.16 (4.12)	30.24 (3.98)	31.29 (6.18)	31.39 (7.83)	39.48 (9.21)	38.95 (9.16)	38.68 (6.58)	38.94 (6.09)	0.09	0.03	0.76	0.00
Body fat (%)	27.11 (7.74)	27.42 (6.87)	25.22 (8.89)	24.75 (8.16)	39.81 (8.03)	38.46 (8.36)	43.24 (7.11)	42.72 (8.18)	29.03 (9.11)	29.70 (7.86)	26.61 (6.70)	27.11 (6.26)	41.51 (6.07)	41.02 (5.96)	42.54 (8.74)	40.87 (8.65)	0.26	0.01	0.65	0.02
Body fat (kg)	16.66 (5.62)	17.01 (5.19)	14.67 (6.30)	14.64 (6.13)	35.00 (9.46)	33.50 (9.57)	40.40 (12.65)	39.67 (13.82)	17.78 (6.69)	19.65 (8.25)	14.67 (6.31)	16.89 (5.60)	37.80 (9.72)	38.15 (9.93)	39.53 (13.44)	37.61 (12.73)	0.45	0.06	0.31	0.01
Skinfolds (mm)																				
Biceps	8.43 (3.95)	8.32 (3.76)	6.87 (2.71)	6.53 (2.41)	22.12 (10.52)	15.46 (15.45)	20.60 (10.54)	15.57 (4.90)	9.04 (4.17)	8.50 (4.00)	7.86 (2.32)	7.82 (2.42)	22.29 (7.93)	15.46 (4.82)	22.85 (9.14)	14.00 (4.53)	0.18	0.01	0.57	0.03
Chest	10.46 (4.97)	11.85 (4.17)	9.53 (3.85)	8.22 (2.65)	25.20 (9.93)	16.25 (3.31)	22.50 (6.19)	17.45 (3.70)	10.77 (4.17)	10.23 (3.93)	12.54 (4.02)	11.95 (5.07)	25.67 (10.84)	17.08 (3.62)	29.90 (9.33)	16.27 (2.94)	0.04 *	0.04	0.10	0.02
Triceps	13.78 (15.17)	14.93 (5.20)	12.69 (5.77)	12.28 (5.23)	27.25 (10.52)	21.87 (7.13)	25.96 (11.02)	21.53 (7.06)	15.18 (4.97)	23.83 (4.79)	16.45 (5.33)	15.59 (5.14)	28.67 (9.60)	23.83 (4.79)	30.54 (9.87)	23.23 (6.77)	0.46	0.06	0.48	0.05
Axillary	7.57 (3.32)	8.14 (3.23)	20.30 (7.47)	6.75 (2.59)	21.17 (8.08)	15.79 (3.63)	20.31 (7.47)	15.19 (3.49)	7.46 (3.30)	16.45 (5.21)	10.09 (4.65)	9.54 (4.54)	20.75 (9.71)	16.45 (5.22)	22.11 (7.47)	16.19 (3.08)	0.69	0.00	0.93	0.00
Abdominal	18.14 (6.79)	21.71 (7.88)	15.81 (4.82)	18.21 (4.87)	43.87 (9.60)	38.79 (5.80)	38.39 (13.12)	35.46 (6.97)	19.64 (5.32)	19.53 (7.07)	22.82 (7.92)	22.36 (7.19)	42.08 (12.02)	35.83 (7.11)	45.31 (16.89)	35.04 (6.97)	0.32	0.01	0.04 *	0.04
Suprailiac	13.11 (3.08)	13.78 (3.42)	11.78 (2.38)	12.18 (2.57)	31.54 (11.59)	23.08 (2.97)	26.54 (9.15)	23.85 (5.09)	13.32 (3.81)	13.57 (3.06)	13.41 (4.43)	13.50 (3.50)	30.91 (14.17)	21.50 (2.74)	31.23 (10.32)	23.00 (5.08)	0.42	0.00	0.22	0.01
Subscapular	13.11 (3.08)	13.78 (3.42)	11.78 (2.38)	12.18 (2.57)	31.54 (11.59)	23.08 (2.97)	26.54 (9.15)	23.84 (5.09)	13.32 (3.81)	13.57 (3.06)	13.41 (4.42)	13.50 (3.50)	30.92 (12.67)	21.50 (2.75)	32.23 (10.32)	23.00 (5.08)	0.43	0.00	0.22	0.01
Thigh	19.68 (6.49)	18.03 (4.69)	18.16 (6.36)	16.84 (6.10)	38.31 (18.80)	26.22 (7.46)	37.15 (14.60)	25.30 (7.00)	20.78 (8.32)	18.10 (5.30)	19.72 (5.27)	19.45 (3.89)	37.62 (13.25)	26.70 (4.73)	42.07 (15.63)	27.77 (6.55)	0.45	0.06	0.86	0.00
Calf	14.28 (5.67)	13.00 (4.53)	26.69 (14.81)	20.73 (6.35)	28.00 (16.00)	18.33 (6.09)	26.69 (14.80)	20.73 (6.35)	13.43 (4.81)	13.50 (5.12)	12.91 (5.12)	12.45 (5.09)	32.5 (13.36)	20.83 (5.13)	32.20 (13.75)	18.12 (5.80)	0.41	0.07	0.23	0.01

Values are expressed in Mean (SD). * Significantly different at *p* < 0.05. U: Untrained; T: Trained; BW: Body weight; BMI: Body Mass Index; WC: Waist circumference; HC: Hip circumference; ES: Effect size.

**Table 3:** Effects on hematological and biochemical parameters before and after 12 weeks of intervention.

	Placebo (*n* = 56)								Gallic Acid (*n* = 50)											
	Eutrophic (U) (*n* = 14)		Eutrophic (T) (*n* = 16)		Obese (U) (*n* = 12)		Obese (T) (*n* = 14)		Eutrophic (U) (*n* = 14)		Eutrophic (T) (*n* = 11)		Obese (U) (*n* = 12)		Obese (T) (*n* = 13)		Supplement × PA × BMI × Time Interaction		Supplement × Time Interaction	
Parameter	Pre	Post	Pre	Post	Pre	Post	Pre	Post	Pre	Post	Pre	Post	Pre	Post	Pre	Post	p	ES	*p*	ES
Blood count																				
Hemoglobin, g/dL	14.50 (1.54)	14.62 (1.49)	13.67 (1.44)	13.92 (1.59)	13.53 (1.63)	13.79 (1.90)	13.16 (1.55)	13.77 (1.38)	14.00 (1.59)	14.12 (1.29)	13.67 (1.44)	14.11 (1.28)	13.33 (1.34)	13.79 (1.93)	13.66 (1.29)	14.25 (1.30)	0.29	0.01	0.71	0.01
Red blood cells (RBC), ×10^6^/µL	4.93 (0.56)	4.75 (0.55)	4.73 (0.37)	4.62 (0.39)	4.98 (0.42)	4.86 (0.53)	4.86 (0.67)	4.81 (0.59)	4.89 (0.49)	4.70 (0.65)	4.63 (0.42)	4.63 (0.43)	4.89 (0.41)	4.83 (0.45)	4.91 (0.56)	4.91 (0.52)	0.65	0.02	0.39	0.08
Hematocrit (%)	42.81 (3.99)	42.04 (4.77)	40.63 (3.45)	42.04 (4.77)	40.42 (4.12)	40.11 (5.22)	40.49 (4.43)	40.48 (3.39)	41.50 (3.73)	40.68 (4.49)	40.19 (2.73)	39.98 (3.30)	40.28 (4.08)	40.72 (4.60)	40.50 (3.33)	41.84 (3.00)	0.99	0.00	0.22	0.01
Neutrophils (%)	51.11 (13.62)	48.26 (13.33)	57.11 (11.48)	53.92 (8.80)	60.26 (8.08)	54.82 (5.63)	62.08 (9.14)	58.32 (7.03)	48.81 (15.56)	48.26 (13.33)	55.91 (9.70)	51.79 (7.23)	61.30 (6.34)	60.22 (3.67)	56.23 (7.93)	50.95 (11.19)	0.95	0.00	0.36	0.09
Lymphocytes (%)	40.10 (12.94)	41.97 (11.90)	33.20 (10.26)	35.90 (8.69)	32.16 (7.30)	36.16 (6.03)	30.64 (8.14)	32.23 (6.75)	36.01 (12.55)	39.91 (10.35)	33.84 (7.46)	36.44 (5.18)	30.72 (5.25)	29.08 (2.56)	35.54 (6.83)	36.10 (7.87)	0.27	0.01	0.43	0.06
Biochemistry																				
Glucose (mg/dL)	79.14 (9.81)	83.14 (7.25)	79.87 (7.06)	81.60 (8.36)	77.25 (10.07)	84.00 (9.41)	81.78 (8.51)	81.07 (11.53)	79.19 (9.45)	84.25 (9.14)	79.90 (7.95)	84.30 (5.58)	84.67 (9.51)	90.25 (15.15)	75.67 (9.09)	77.75 (11.62)	0.54	0.04	0.79	0.01
HbA1c (%)	4.69 (0.67)	5.15 (0.32)	4.26 (0.84)	5.02 (0.22)	4.38 (0.46)	5.15 (0.33)	4.32 (0.43)	5.02 (0.22)	4.63 (0.62)	4.99 (0.28)	4.96 (0.60)	5.16 (0.23)	4.11 (0.59)	5.15 (0.32)	3.98 (0.46)	4.55 (0.90)	0.99	0.00	0.85	0.00
Insulin (µIU/mL)	6.21 (2.65)	7.56 (3.17)	5.31 (2.52)	5.55 (1.93)	11.62 (5.07)	11.22 (5.92)	14.25 (8.70)	9.30 (3.96)	5.49 (3.03)	6.43 (2.75)	4.52 (1.83)	6.45 (2.96)	15.13 (5.30)	15.50 (8.35)	10.61 (3.69)	10.73 (4.90)	0.53	0.00	0.04 *	0.04
Urea (mg/dL)	25.79 (5.95)	28.14 (10.48)	24.50 (7.94)	23.62 (5.73)	28.00 (10.42)	25.17 (6.96)	26.50 (8.51)	25.93 (6.68)	26.43 (6.82)	26.93 (5.50)	28.00 (7.32)	28.82 (5.27)	25.00 (9.04)	25.08 (7.14)	24.50 (5.52)	26.25 (7.27)	0.50	0.07	0.41	0.07
Creatinine (mg/dL)	0.92 (0.25)	0.95 (0.22)	0.83 (0.23)	0.93 (0.18)	0.90 (0.21)	0.86 (0.24)	1.01 (0.17)	0.84 (0.17)	0.89 (0.19)	0.95 (0.16)	0.94 (0.21)	1.02 (0.29)	0.91 (0.12)	0.75 (0.18)	0.97 (0.13)	0.79 (0.22)	0.43	0.06	0.49	0.05
Albumin (g/dL)	4.73 (0.33)	4.63 (0.42)	4.65 (0.22)	4.52 (0.28)	4.26 (0.54)	4.29 (0.42)	4.53 (0.32)	4.16 (0.85)	4.70 (0.25)	4.47 (0.53)	4.67 (0.45)	4.76 (0.36)	4.46 (0.29)	4.63 (0.42)	4.32 (0.42)	4.55 (0.36)	0.80	0.01	0.04 *	0.04
AST (U/L)	26.57 (28.26)	21.21 (9.39)	18.47 (4.01)	18.47 (7.20)	19.25 (4.97)	23.83 (11.01)	19.57 (6.33)	20.50 (6.03)	19.27 (6.06)	18.63 (5.16)	34.18 (42.15)	24.54 (13.45)	23.92 (8.12)	24.67 (9.83)	19.61 (3.01)	20.92 (2.69)	0.15	0.02	0.52	0.04
ALT (U/L)	23.93 (32.10)	18.78 (12.42)	14.60 (5.46)	13.33 (3.83)	23.91 (12.09)	40.08 (43.11)	24.57 (17.05)	23.64 (14.08)	24.83 (28.95)	19.92 (16.96)	26.72 (17.70)	26.90 (17.05)	37.00 (18.36)	34.08 (20.08)	24.85 (12.69)	28.46 (15.14)	0.19	0.01	0.46	0.06
Uric acid, mg/dL	4.15 (1.92)	4.11 (1.44)	3.38 (0.90)	3.46 (1.35)	4.45 (1.92)	4.96 (1.78)	4.41 (1.47)	4.81 (1.62)	3.70 (1.19)	3.69 (1.29)	3.89 (1.42)	4.02 (1.90)	4.88 (1.59)	5.63 (1.40)	4.94 (1.77)	5.88 (1.58)	0.79	0.01	0.40	0.07
Lipid level (mg/dL)																				
Cholesterol total	168.85 (34.38)	160.86 (22.48)	152.69 (43.22)	146.62 (50.78)	207.5 (45.86)	197.00 (42.79)	192.14 (30.53)	187.25 (46.42)	158.25 (24.25)	162.92 (25.28)	152.69 (43.23)	196.09 (38.03)	182.17 (50.50)	179.00 (45.97)	174.38 (62.03)	171.69 (37.78)	0.79	0.01	0.09	0.03
HDL	68.08 (27.70)	59.69 (21.74)	62.25 (15.75)	57.87 (15.09)	46.42 (13.62)	42.25 (13.48)	53.14 (15.15)	52.28 (16.42)	57.92 (11.28)	58.08 (11.73)	63.81 (13.12)	58.45 (12.25)	50.75 (11.28)	50.41 (11.73)	47.25 (15.04)	47.29 (12.27)	0.65	0.02	0.24	0.01
LDL	86.28 (33.38)	81.07 (23.91)	76.04 (28.71)	75.37 (37.93)	134.43 (42.96)	12242 (3439)	113.07 (22.25)	113.28 (33.61)	85.85 (22.21)	89.83 (28.24)	107.80 (35.22)	117.54 (36.89)	108.17 (32.46)	103.67 (33.46)	104.50 (52.92)	104.58 (37.61)	0.61	0.03	0.12	0.025
Triglycerides	76.36 (27.72)	87.71 (31.78)	73.19 (43.73)	71.50 (24.10)	132.92 (62.97)	175.67 (89.26)	127.86 (58.89)	111.26 (78.99)	68.91 (27.03)	87.45 (36.79)	90.36 (43.09)	147.67 (75.20)	123.25 (62.08)	147.67 (75.20)	121.53 (54.20)	152.69 (75.29)	0.09	0.02	0.14	0.02

Values are expressed in Mean (SD). * Significantly different at *p* < 0.05.

## Data Availability

The authors confirm that the data and materials supporting the findings of this study are available within the article.

## Supplementary Materials

The following supporting information can be downloaded at: https://www.mdpi.com/article/10.3390/nu18020311/s1, Table S1: Sensitivity analysis including the excluded participant. Table S2: Between-group differences in body composition after 12 weeks of intervention estimated by ANCOVA (reference: Eutrophic Untrained Placebo). Table S3: Between-group differences on hematological and biochemical parameters after 12 weeks of intervention estimated by ANCOVA (reference: Eutrophic Untrained Placebo).

## Abbreviations

AMPK	AMP-activated protein kinase
SIRT1	Sirtuin 1
BMI	Body Mass Index
ICF	Informed Consent Form
TEE	Total Energy Expenditure
WHR	Waist-to-hip
HbA1c	Glycated hemoglobin
ALT	Aminotransferase
AST	Aspartate transaminase
HDL-c	High-density lipoprotein
LDL-c	Low-density lipoprotein
TG	Triglycerides
IQR	Interquartile range
GLP-1	Glucagon-like peptide-1
GIP	Insulinotropic polypeptide
