# Supplementary material for: Combined Effects of Gallic Acid Supplementation and Physical Training on Body Composition and Biochemical Parameters in Obese Patients: A Randomized, Double-Blinded, Placebo-Controlled Clinical Trial
Source: Nutrients. 2026 Jan 19;18(2):311. doi: 10.3390/nu18020311 (PMC12845335; doi:10.3390/nu18020311)
Supplement: Supplementary file 1 [file nutrients-18-00311-s001.zip › nutrients-4061362-supplementary.pdf]

Supplementary Materials

During data analysis, one participant showed a marked increase in biochemical parameters (blood glucose, glycated hemoglobin, and insulin) during follow-up. These values were identified as extreme outliers using predefined criteria. Sensitivity analyses including this participant were performed and showed no significant changes in the overall direction or significance of the results, as shown in Table S1.

Table S1. Sensitivity analysis including the excluded participant

	With outlier (N = 107)				Without outlier (N = 106)			
**Parameter**	Supplement X PA X BMI X Time interaction		Supplement X Time interaction		Supplement X PA X BMI X Time interaction		Supplement X Time interaction	
	p	ES	*p*	ES	*P*	ES	*P*	ES
Glucose (mg/dL)	0.50	0.00	0.89	0.00	0.54	0.04	0.79	0.01
HbA1c (%)	0.93	0.00	0.92	0.00	0.99	0.00	0.85	0.00
Insulin (µIU/mL)	0.58	0.00	0.04*	0.04	0.53	0.00	0.04*	0.04

* Significantly different at p < 0,05

Table S2: Between-group differences in body composition after 12 weeks of intervention estimated by ANCOVA (reference: Eutrophic Untrained Placebo)

	Eutrophic (TP) (N = 16)	Obese(UP) (N = 12)	Obese (TP) (N = 14)	Eutrophic (UGA) (N = 14)	Eutrophic (TGA) (N = 11)	Obese (UGA) (N = 12)	Obese (TGA) (N = 13)
**Anthropometry**							
BW(Kg)							
BMI (kg/m²)	0.17 (−3.12; 3.46) $p = 1.000$	1.30 (-2.66; 5.26) $p = 1.000$	2.44 (-1.59; 6.48) $p = 1.000$	-0.12 (−3.51; 3.27) $p = 1.000$	0.09 (−3.52; 3.52) $p = 1.000$	0,58 (-3.49; 4.66) $p = 1.000$	1.25 (-2.79; 5.30) $p = 1.000$
WC (cm)	0.03 (−1.25; 1,30) $p = 1.000$	0.56 (-1,02; 2.30) $p = 1.000$	0.70 (-1,00; 2.40) $p = 1.000$	0.12 (−1.2; 1.44) $p = 1.000$	0.9 (−0.3; 2.1) $p = 0.148$	-0,04 (-1.44; 1.37) $p = 1.000$	0.64 (-1,02; 2.30) $p = 1.000$
HC (cm)	0.35 (−3.15; 3.86) $p = 1.000$	-3,11 (−4.51; 3,89) $p = 1.000$	-0.59 (−4.95; 3.77) $p = 1.000$	1.04 (−2.64; 4,72) $p = 1.000$	1.67 (−2.18; 5.52) $p = 1.000$	-0.87 (−5.27; 3.53) $p = 1.000$	-0.19 (−4.63; 4.25) $p = 1.000$
Waist-to-hip ratio	0.02 (−0.03; 0.07) $p = 1.000$	0.01 (−0.06; 0.06) $p = 1.000$	0.01 (−0.06; 0.04) $p = 1.000$	-0.01 (−0.06; 0.04) $p = 1.000$	0.01 (−0.05; 0.06) $p = 1.000$	-0.02 (−0.08; 0.03) $p = 1.000$	-0.03 (−0.09; 0.02) $p = 1.000$
**Body Composition**							
Fat-free mass (%)	-0.98 (−4.34; 2.37) $p = 1.000$	-0.36 (−4.25; 3.53) $p = 1.000$	0.88 (−3.05; 4.81) $p = 1.000$	0.56 (−2.90; 4.03) $p = 1.000$	0.13 (−3.55; 3.82) $p = 1.000$	1.12 (−2.85; 5.09) $p = 1.000$	-0.39 (−4.34; 3.56) $p = 1.000$
Fat-free mass (kg)	-1.06 (−3.67; 1.54) $p = 1.000$	-0.88 (−3.76; 2.00) $p = 1.000$	0.24 (−2.53; 3.01) $p = 1.000$	-0.55 (−3.25; 2.14) $p = 1.000$	-0.64 (−3.51; 2.22) $p = 1.000$	-0.67 (−3.56; 2.22) $p = 1.000$	-1.77 (−4.59; 1.04) $p = 1.000$
Skeletal muscle mass (%)	0.54 (−6.06; 7.15) $p = 1.000$	-1.33 (−8.95; 6.95) $p = 1.000$	2.83 (−4.67; 10.33) $p = 1.000$	0.62 (−6.21; 7.45) $p = 1.000$	-1.36 (−6.06; 7.15) $p = 1.000$	2.77 (−4.84; 10.37) $p = 1.000$	1.95 (−5.58; 9.48) $p = 1.000$
Body water (%)	1.09 (−3.91; 1.74) $p = 1.000$	-0.40 (−3.65; 2.85) $p = 1.000$	0.30 (−2.96; 3.56) $p = 1.000$	0.01 (−2.92; 2.94) $p = 1.000$	-0.11 (−3.22; 3.00) $p = 1.000$	0.61 (−2.68; 3.90) $p = 1.000$	-0.44 (−3.71; 2.83) $p = 1.000$
Body water (kg)	-0.54 (−2.61; 1.52) $p = 1.000$	-0.16 (−2.44; 2.12) $p = 1.000$	0.85 (−1.37; 3.06) $p = 1.000$	-0.12 (−2.25; 2.01) $p = 1.000$	-0.14 (−2.40; 2.13) $p = 1.000$	0.38 (−1.94; 2.70) $p = 1.000$	-0.40 (−2.65; 1.85) $p = 1.000$
Body fat (%)	0.98 (−2.41; 4.37) $p = 1.000$	0.31 (−3.61; 4.24) $p = 1.000$	-0.89 (−4.86; 3.09) $p = 1.000$	-0.57 (−4.06; 2.93) $p = 1.000$	-0.14 (−3.86; 3.58) $p = 1.000$	-0.74 (−4.75; 3.27) $p = 1.000$	0.34 (−3.65; 4.33) $p = 1.000$
Body fat (kg)	0.48 (−3.68; 4.63) $p = 1.000$	1.02 (−4.00; 6.03) $p = 1.000$	0.01 (−5.21; 5.22) $p = 1.000$	-1.57 (−5.86; 2.71) $p = 1.000$	-0.17 (−4.73; 4.40) $p = 1.000$	-0.96 (−6.15; 4.23) $p = 1.000$	1.22 (−4.00; 6.44) $p = 1.000$
**Skinfolds**							
Biceps (mm)	1.27 (−2.52; 5.07) $p = 1.000$	-2.57 (−7.12; 1.98) $p = 1.000$	-3.19 (−7.50; 1.12) $p = 1.000$	0.02 (−3.89; 3.94) $p = 1.000$	0.31 (−3.85; 4.48) $p = 1.000$	-4.14 (−8.70; 0.42) $p = 1.000$	-0.87 (−5.40; 3.65) $p = 1.000$

	Eutrophic (TP) (N = 16)	Obese(UP) (N = 12)	Obese (TP) (N = 14)	Eutrophic (UGA) (N = 14)	Eutrophic (TGA) (N = 11)	Obese (UGA) (N = 12)	Obese (TGA) (N = 13)
Triceps (mm)	2.13 (−3.18; 7.44)<br>*p* = 1.000	-0.60 (−6.80; 5.61)<br>*p* = 1.000	-0.87 (−6.88; 5.14)<br>*p* = 1.000	-0.13 (−5.61; 5.35)<br>*p* = 1.000	0.60 (−5.26; 6.46)<br>*p* = 1.000	-1.89 (−8.20; 4.43)<br>*p* = 1.000	-0.40 (−6.77; 5.96)<br>*p* = 1.000
Axillary (mm)	1.26 (−2.82; 5.33)<br>*p* = 1.000	-4.72 (−9.77; 0.32)<br>*p* = 0.094	-4.31 (−9.20; 0.58)<br>*p* = 0.157	0.37 (−3.84; 4.58)<br>*p* = 1.000	-0.86 (−5.37; 3.65)<br>*p* = 1.000	-5.48 (−10.49; -0.47)<br>***p* = 0.019***	-4.92 (−9.97; 0.13)<br>*p* = 0.065
Abdominal (mm)	2.70 (−4.46; 9.86)<br>*p* = 1.000	-8.30 (−17.46; 0.85)<br>*p* = 0.124	-6.85 (−15.20; 1.51)<br>*p* = 0.276	2.69 (−4.70; 10.07)<br>*p* = 1.000	0.94 (−6.98; 8.86)<br>*p* = 1.000	-5.96 (−14.93; 3.01)<br>*p* = 0.991	-4.06 (−13.24; 5.11)<br>*p* = 1.000
Suprailiac (mm)	3.21 (−1.97; 8.38)<br>*p* = 1.000	-2.64 (−9.02; 3.74)<br>*p* = 1.000	-5.10 (−11.36; 1.17)<br>*p* = 0.290	1.70 (−3.60; 7.02)<br>*p* = 1.000	1.48 (−4.18; 7.14)<br>*p* = 1.000	-3.28 (−9.91; 3.35)<br>*p* = 1.000	-4.89 (−11.42; 1.64)<br>*p* = 0.506
Subscapular (mm)	1.35 (−2.59; 5.30)<br>*p* = 1.000	-5.92 (−10.81; -1.03)<br>***p* = 0.005***	-7.60 (−12.11; -3.08)<br>***p* = 0.000***	0.25 (−3.82; 4.32)<br>*p* = 1.000	0.34 (−3.40; 4.68)<br>*p* = 1.000	-4.45 (−9.30; 0.40)<br>*p* = 0.112	-5.89 (−10.69; -1.10)<br>***p* = 0.004***
Thigh (mm)	0.76 (−4.92; 6.45)<br>*p* = 1.000	-2.94 (−9.68; 3.80)<br>*p* = 1.000	-2.35 (−8.77; 4.07)<br>*p* = 1.000	0.24 (−5.63; 6.11)<br>*p* = 1.000	-1.40 (−7.66; 4.85)<br>*p* = 1.000	-3.62 (−10.18; 2.95)<br>*p* = 1.000	-3.42 (−10.12; 3.27)<br>*p* = 1.000
Calf (mm)	1.36 (−3.62; 6.34)<br>*p* = 1.000	-1.12 (−6.75; 4.52)<br>*p* = 1.000	-3.91 (−9.40; 1.56)<br>*p* = 0.667	-0.76 (−5.90; 4.38)<br>*p* = 1.000	0.12 (−5.36; 5.60)<br>*p* = 1.000	-2.21 (−8.06; 3.64)<br>*p* = 1.000	0.39 (−5.50; 6.22)<br>*p* = 1.000

Values are presented as adjusted mean differences (β) with 95% confidence intervals (95% CI). p values refer to comparisons between each group and the reference group. UP: untrained placebo; TP: trained placebo; UGA: untrained gallic acid; TGA: trained gallic acid; WC: Waist circumference; HC: Hip circumference; *p value < 0.05 was considered statistically significant.

Table S3: Between-group differences on hematological and biochemical parameters after 12 weeks of intervention estimated by ANCOVA (reference: Eutrophic Untrained Placebo).

	**Eutrophic (TP) (N = 16)**	**Obese(UP) (N = 12)**	**Obese (TP) (N = 14)**	**Eutrophic (UGA) (N = 14)**	**Eutrophic (TGA) (N = 11)**	**Obese (UGA) (N = 12)**	**Obese (TGA) (N = 13)**
**Blood count**							
Hemoglobin, g/dL	-0.07 (−1.00; 0.86) *p* = 1.000	-0.07 (−1.07; 9.36) *p* = 1.000	-0.39 (−1.37; 0.59) *p* = 1.000	0.24 (−0.72; 1.19) *p* = 1.000	-0.35 (−1.37; 0.68) *p* = 1.000	-0.20 (−1.21; 0.81) *p* = 1.000	-0.41 (−1.39; 0.57) *p* = 1.000
Red blood cells (RBC), ×10^6/µL	-0.05 (−0.39; 0.28) *p* = 1.000	-0.08 (−0.44; 0.28) *p* = 1.000	-0.13 (−0.48; 0.22) *p* = 1.000	0.00 (−0.34; 0.35) *p* = 1.000	-0.15 (−0.52; 0.23) *p* = 1.000	-0.15 (−0.52; 0.22) *p* = 1.000	-0.18 (−0.53; 0.17) *p* = 1.000
Hematocrit (%)	0.30 (−2.86; 3.46) *p* = 1.000	-0.09 (-3.50; 3.30) *p* = 1.000	-0.41 (-3.68; 2.85) *p* = 1.000	0.25 (−2.99; 3.50) *p* = 1.000	-0.16 (-3.65; 3.32) *p* = 1.000	-0.83 (-4.23; 2.57) *p* = 1.000	-1.76 (-5.08; 1.57) *p* = 1.000
Leukocytes (mm^3)	370.65 (−2684.63; 1943.33) *p* = 1.000	-0.07 (−1.00; 0.86) *p* = 1.000					
Neutrophils (%)	-2.99 (−12.56; 6.57) *p* = 1.000	-2.49 (−12.90; 7.91) *p* = 1.000	-5.19 (−15.49; 5.11) *p* = 1.000	-4.13 (−13.91; 5.65) *p* = 1.000	-1.40 (−11.87; 9.07) *p* = 1.000	-7.43 (−18.41; 3.54) *p* = 1.000	-0.42 (−10.45; 9.61) *p* = 1.000
Lymphocytes (%)	2.34 (−5.31; 9.99) *p* = 1.000	1.52 (−6.73; 9.76) *p* = 1.000	4.61 (−3.40; 12.64) *p* = 1.000	-0.15 (−7.95; 7.65) *p* = 1.000	2.14 (−6.23; 10.51) *p* = 1.000	7.82 (−0.69; 16.33) *p* = 0.111	3.40 (−4.55; 11.36) *p* = 1.000
**Biochemistry**							
Glucose (mg/dL)	0.63 (−10.91; 12.14) *p* = 1.000	-1.63 (−13.82; 10.56) *p* = 1.000	3.15 (−8.58; 14.89) *p* = 1.000	0.55 (−11.14; 12.25) *p* = 1.000	-0.85 (−13.66; 11.97) *p* = 1.000	-4.85 (−17.18; 7.48) *p* = 1.000	3.97 (−8.27; 16.21) *p* = 1.000
HbA1c (%)	-0.09 (−0.63; 0.45) *p* = 1.000	-0.31 (−0.88; 0.25) *p* = 1.000	-0.19 (−0.76; 0.37) *p* = 1.000	-0.12(−0.66; 0.42) *p* = 1.000	-0.23 (−0.81; 0.34) *p* = 1.000	-0.58 (−1.16; -0.01) ***p* = 0.043***	0.21 (−0.37; 0.80) *p* = 1.000
Insulin (µIU/mL)	1.51 (−3.10; 6.12) *p* = 1.000	-0.68 (−5.83; 4.47) *p* = 1.000	2.69 (−2.54; 7.92) *p* = 1.000	0.72 (−4.12; 5.57) *p* = 1.000	-0.10 (−5.67; 5.46) *p* = 1.000	-3.02 (−8.53; 2.48) *p* = 1.000	-0.75 (−5.73; 4.24) *p* = 1.000
Urea (mg/dL)	4.00 (−3.56; 11.56) *p* = 1.000	3.87 (−4.27; 12.01) *p* = 1.000	2.50 (−5.30; 10.30) *p* = 1.000	1.47 (−6.33; 9.27) *p* = 1.000	0.22 (−8.12; 8.55) *p* = 1.000	2.74 (−5.38; 10.86) *p* = 1.000	1.37 (−6.75; 9.50) *p* = 1.000
Creatinine (mg/dL)	0.01 (−0.23; 0.25) *p* = 1.000	0.09 (−0.17; 0.35) *p* = 1.000	0.13 (−0.13; 0.38) *p* = 1.000	-0.01 (−0.27; 0.23) *p* = 1.000	-0.06 (−0.33; 0.20) *p* = 1.000	0.21 (−0.05; 0.47) *p* = 0.323	0.18 (−0.08; 0.43) *p* = 0.817
Albumin (g/dL)	0.06 (−0.48; 0.61) *p* = 1.000	0.11 (−0.71; 0.48) *p* = 1.000	0.37 (−0.21; 0.95) *p* = 1.000	0.18 (−0.38; 0.74) *p* = 1.000	-0.16 (−0.76; 0.44) *p* = 1.000	-0.14 (−0.73; 0.46) *p* = 1.000	-0.11 (−0.71; 0.48) *p* = 1.000
AST (U/L)	1.11 (−8.19; 10.41) *p* = 1.000	-4.10 (−13.93; 5.73) *p* = 1.000	-0.70 (−10.14; 8.74) *p* = 1.000	1.28 (−8.34; 10.91) *p* = 1.000	-1.79 (−11.86; 8.27) *p* = 1.000	-3.99 (−13.77; 5.79) *p* = 1.000	-1.11 (−10.73; 8.50) *p* = 1.000

	Eutrophic (TP) (N = 16)	Obese(UP) (N = 12)	Obese (TP) (N = 14)	Eutrophic (UGA) (N = 14)	Eutrophic (TGA) (N = 11)	Obese (UGA) (N = 12)	Obese (TGA) (N = 13)
ALT (U/L)	1.62 (−20.44; 23.68) *p* = 1.000	-21.30 (−44.46; 1.86) *p* = 0.110	-4.59 (−26.85; 17.66) *p* = 1.000	-0.60 (−22.85; 21.65) *p* = 1.000	-6.97 (−30.71; 16.76) *p* = 1.000	-9.93 (−33.43; 13.57) *p* = 1.000	-9.29 (−31.97; 13.38) *p* = 1.000
Uric acid, mg/dL	0.15 (−1.28; 1.58) *p* = 1.000	-0.64 (−2.17; 0.88) *p* = 1.000	-0.53 (−2.03; 0.96) *p* = 1.000	0.13 (−1.36; 1.63) *p* = 1.000	-0.07 (−1.63; 1.549) *p* = 1.000	-1.04 (−2.58; 0.49) *p* = 0.869	-1.26 (−2.77; 0.24) *p* = 0.235
**Lipid level (mg/dl)**							
Cholesterol total (mg/dl)	1.98 (−26.50; 30.45) *p* = 1.000	-6.86 (−38.13; 24.40) *p* = 1.000	-8.78 (−38.34; 20.78) *p* = 1.000	-7.05 (−36.31; 22.21) *p* = 1.000	-19.62 (−51.03; 11.78) *p* = 1.000	-8.06 (−38.60; 22.48) *p* = 1.000	-6.65 (−36.47; 23.18) *p* = 1.000
HDL (mg/dl)	-2.62 (−13.71; 8.47) *p* = 1.000	0.96 (−11.52; 13.44) *p* = 1.000	-3.96 (−15.67; 7.75) *p* = 1.000	-6.72 (−18.27; 4.84) *p* = 1.000	-2.00 (−14.14; 10.13) *p* = 1.000	-3.91 (−16.16; 8.34) *p* = 1.000	-0.32 (−12.75; 12.11) *p* = 1.000
LDL (mg/dl)	-2.09 (−26.76; 22.59) *p* = 1.000	-4.69 (−32.65; 23.26) *p* = 1.000	-11.82 (−37.71; 14.0) *p* = 1.000	-6.16 (−31.56; 19.23) *p* = 1.000	-20.09 (−47.39; 7.21) *p* = 0.561	-5.93 (−32.63; 20.77) *p* = 1.000	-9.64 (−36.24; 16.96) *p* = 1.000
Triglycerides (mg/ dl)	13.84 (−34.68; 62.38) *p* = 1.000	-45.72 (−99.45; 8.00) *p* = 0.208	14.88 (−36.58; 66.34) *p* = 1.000	-0.17 (−51.26; 50.92) *p* = 1.000	-5.10 (−58.62; 48.42) *p* = 1.000	-24.94 (−78.18; 28.30) *p* = 1.000	-31.24 (−83.34; 20.85) *p* = 1.000

Values are presented as adjusted mean differences (β) with 95% confidence intervals (95% CI). p values refer to comparisons between each group and the reference group. UP = untrained placebo; TP = trained placebo; UGA = untrained gallic acid; TGA = trained gallic acid. *p value < 0.05 was considered statistically significant.
